# Period doubling induced by thermal noise amplification in genetic circuits

**DOI:** 10.1038/srep07088

**Published:** 2014-11-18

**Authors:** G. Ruocco, A. Fratalocchi

**Affiliations:** 1 PRIMALIGHT, Faculty of Electrical Engineering; Applied Mathematics and Computational Science, King Abdullah University of Science and Technology (KAUST), Thuwal 23955-6900, Saudi Arabia; 2 Sapienza University of Rome, Department of Physics, Piazzale Aldo Moro 5, 00185 Rome, Italy; 3 Center for Life Nano Science@Sapienza, Istituto Italiano di Tecnologia, Viale Regina Elena 291, 00161, Rome, Italy

## Abstract

Rhythms of life are dictated by oscillations, which take place in a wide rage of biological scales. In bacteria, for example, oscillations have been proven to control many fundamental processes, ranging from gene expression to cell divisions. In genetic circuits, oscillations originate from elemental block such as autorepressors and toggle switches, which produce robust and noise-free cycles with well defined frequency. In some circumstances, the oscillation period of biological functions may double, thus generating bistable behaviors whose ultimate origin is at the basis of intense investigations. Motivated by brain studies, we here study an “elemental” genetic circuit, where a simple nonlinear process interacts with a noisy environment. In the proposed system, nonlinearity naturally arises from the mechanism of cooperative stability, which regulates the concentration of a protein produced during a transcription process. In this elemental model, bistability results from the coherent amplification of environmental fluctuations due to a stochastic resonance of nonlinear origin. This suggests that the period doubling observed in many biological functions might result from the intrinsic interplay between nonlinearity and thermal noise.

The diversity of multicellular organisms is regulated by a system of rhythms that affect all forms of their functionalities^1^,^2^,^3^. A feature shared by many organic systems, however, is the period doubling of their internal cycles. Appearing spontaneously or in response to external disturbances, the cycle of cellular organisms is observed to become more complex, enriching its activity and leading the system to complete its functionality with a period that is two times larger than initial one^4^,^5^,^6^,^7^,^8^,^9^,^10^,^11^. This phenomenon is observed in a wide range of scales and can be found, for example, in the arrhythmia of cardiac functions^4^, or in the firing of neurons^5^,^6^, where it has been tentatively explained on the basis of the well know bifurcation to chaos paradigm^9^,^10^, or in the distribution of population growth in yeast^12^, where it was ascribed to genetic regulatory circuits. Period doubling has also been observed during cell divisions and circadian cycles, where it can manifest spontaneously^11^ or in response to chemical perturbations^13^. Similarly, it has been shown that the genome-wide transcriptional oscillation in yeast can experience period doubling in reaction to drugs^14^. The ubiquity of this phenomenon challenges the stability of the “biological oscillators”^15^,^16^, and still deserves a comprehensive and clear interpretation.

In this paper, we investigate the doubling process starting from an elementary genetic circuit that combines two ubiquitous aspects of biosystems: inhibitory mechanisms and noise. The active role of noise in genetic and biochemical circuits has already been emphasized^17^,^18^,^19^,^20^,^21^,^22^ and its amplification has been recently found at the basis of novel and important physical phenomena^23^. On the other hand, the importance of inhibitory functions has already been recognized in neurobiology, where they originate nonlinear networks that are at the basis of a diversity of brain functionalities^24^,^25^,^26^. We demonstrate that our elemental genetic circuit can be mapped on an electric RLCD series, made by a resistor, an inductance, a capacitor and a diode, which represent a general model of resonant wave interactions in the presence of a strongly nonlinear response. In this elementary model, nonlinearity arises from the inhibitory function of the diode, which regulates its voltage (i.e., the protein concentration in the genetic counterpart). We show that the two aspects of the genetic dynamics, namely nonlinearity and noise, do not produce any interesting evolution if considered alone. On the contrary, when noise and nonlinearity are combined together, period doubling appears. Analytic theory demonstrates that such phenomenon is sustained from nonlinear parametric resonance, which strongly amplifies noise in a narrow band that resonates with the coherent part of environmental fluctuations. This suggest that period doubling commonly observed in biological activity can be ascribed to the interplay between nonlinearity and noise.

## Results

### From genetic models to nonlinear electric circuits

**Figure 1:**
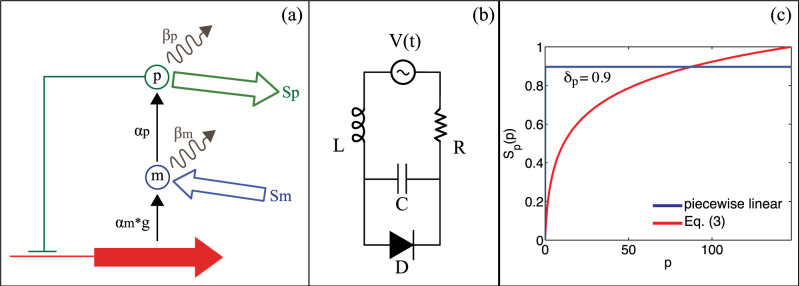
Functional autorepressor scheme. (a) Genetic and (b) equivalent RLD circuit. Panel (c) shows the nonlinear protein degradation function (solid line) and its piecewise linear approximation in the case of *χ_t_* = 0.2 (dashed line).

We consider a simple protein transcription process described by the functional autorepressor^27^ sketched in Fig. 1a, with its associated dynamics for the protein *p*(*t*) and mRNA *m*(*t*) concentrations, respectively^28^,^29^: 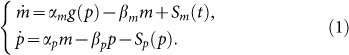
In Eqs. (1), *α_i_* and *β_i_* stand for the reaction and degradation rates, respectively, while *g*(*p*) is the promoter-inhibitor activity function. The latter models the mechanism where the gene product represses its own transcription. The function *g* is an upper bounded decreasing function of *p*^30^, which can be expanded in series: 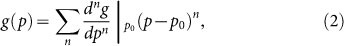
with *p*_0_ a constant parameter. We begin our discussion by considering the simplest case given by a first order function *g*(*p*) = (*p*_0_ − *p*), and then discuss the effects of high order terms (see the Discussion Section). Reaction/degration rates are complemented by the presence of an environment at nonzero temperature, modeled by the time dependent terms *S_p_*[*p*(*t*)] and *S_m_*(*t*). The function *S_p_*, in particular, accounts for an additional protein concentration depletion due to its consumption in the different cell compartments, while *S_m_*(*t*) models the action of the environment on the mRNA transcription rate^2^. In order to pursue a general theory, we decompose *S_m_* into two main contributions *S_m_*(*t*) = *σ*(*t*) + *η*(*t*). The term *σ*(*t*) represents the coherent (periodic) contribution originating from cyclic mechanisms (such as e.g. circadian rhythms and/or cell divisions), while *η*(*t*) models the incoherent (noise) part arising from random fluctuations. The fluctuations arising from *S_m_*, due to the coupling between *m* and *p*, provide a statistical noise source also for the protein concentration *p*. We finally model the term *S_p_* by a nonlinear *p* dependent degradation function, which we express in the simple form: 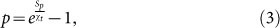
being *χ_p_* the nonlinear degradation rate of the protein concentration. The nonlinear response described by Eq. (3) is illustrated in Fig. 1c (solid line) and models in a simple fashion the mechanism of cooperative stability, which is widely observed in many experiments^31^,^32^ and leads to an enhanced degradation rate at low protein concentration and a lower depletion at higher concentration^33^. Equation (3) has the dynamics of a “biological” diode, which leads to an interesting equivalent electric circuit representation. In order to illustrate the circuit analogy within the simplest theoretical framework, we model the exponential response in Eq. (3) by a piecewise linear model (Fig. 1c dashed line). This type of approximation is largely employed in electronic circuits to provide a simple yet accurate representation of nonlinear components such as diodes and transistors (see e.g., chapter 3 of^34^). The piecewise linear model is characterized as follows: 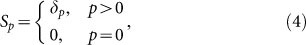
with a constant *δ_p_* estimated from (3): 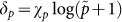, being 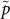 the characteristic scale for the protein concentration in the autoregressor dynamics. For the situation illustrated in Fig. 1c with *χ_p_* = 0.2, if we assume a characteristic concentration 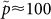 we obtain *δ_p_* = 0.9. Equations (4) generate the following equations of motion when the protein concentration reaches *p* = 0 (OFF state): 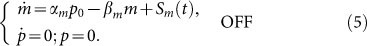
where only the mRNA *m*(*t*) is allowed to evolve while the protein *p* concentration stays in its minimum (zero) value. The duration of this state depends on the value of the time derivative 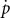 in Eq. (1). When the latter is negative, and therefore showing a tendency of the system to decrease the protein concentration below the minimum, the dynamics is maintained in the OFF condition. When 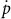 becomes positive, conversely, the system gets back to the ON state and the concentration *p* increases again. From the second of Eqs. (1), we immediately observe that the threshold for a positive protein production, when *p* = 0, is represented by *α_p_m*(*t*) = *S_p_* (i.e., when *α_p_m*(*t*) ≥ *S_p_* we have 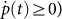. It is worthwhile emphasizing that such ON-OFF states provide a piecewise linear representation for Eq. (3) and do not generate any discontinuity in the bio-physical system, which follows the continuous dynamics described by Eqs. (1) – (3) at every instant. To map Eqs. (1) – (5) into a simple nonlinear electric circuit, we introduce the following linear change of variables (*p*, *m*) → (*x*, *y*), being (*x*, *y*) charge and current, respectively, of the RLCD circuit of Fig. 1b with: 
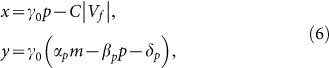
with *V_f_* and *C* the forward bias and the junction capacity of the diode, respectively. The circuit constants (*R*, *L*, *C*, *V_f_*, *V*(*t*)) and the dimensionality scaling constant *γ*_0_ are related to the autorepressor parameters by: 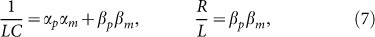

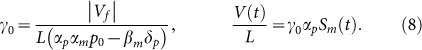


Under the coordinates change (6) – (7), the functional autorepressor (1) – (5) is mapped into the equivalent electric dynamics: 
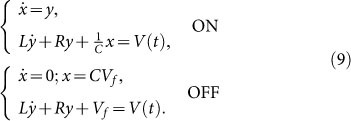
with *V_d_*(*t*) = *V_f_* when the diode is in the OFF state and 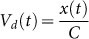 when the diode is conducting (ON state). Switching conditions are promptly interpreted from (6) as the conduction/not conduction states of the diode, and read OFF→ON when *y* > 0 and ON→OFF when *x* = *CV_f_*. These are equivalent to the conditions *α_p_m* > *δ_p_* and *p* = 0 of the genetic circuit (1) – (5). To account for the full nonlinear form of the protein depletion function in Eq. (3), we need to consider the exponential nonlinear response of the diode in the circuit of Fig. 1c, thus obtaining: 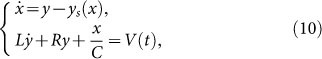
with: 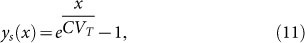
being *y_s_* the current flowing into the diode and *V_T_* its equivalent thermal voltage. The latter can be estimated by inverting Eq. (11), obtaining 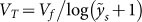, with 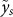 the characteristic current flowing into the diode. For a typical current *y_s_* ≈ 1*mA* and a potential *V_f_* = |*x*|/*C* ≈ 0.6 *V*, we have *V_T_* ≈ 600 *V*. The protein nonlinear depletion rate *χ_p_* characterizes the equivalent thermal voltage *V_T_* of the diode through Eqs. (7) that, in turn, define the diode potential *V_f_*. Equations (10) – (11) constitute the equivalent electric circuit of Eqs. (1) and (3), with Eq. (11) being the electric counterpart of (3). Equations (5) and (9), conversely, represent the corresponding piecewise linear models. Given a set of “genetic” parameters *α_i_*, *β_i_*, *S_i_*, the equivalent electric system possesses two free constants: *L* and *V_f_*. While the diode bias *V_f_* defines the current amplitude scale, the inductance *L* provides an arbitrary scale for the resonant frequency 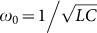 and quality factor *Q* = *ω*_0_*L*/*R* of the circuit when the diode is conducting. Besides the present context where the RLCD circuit has been derived, we observe that Eqs. (10) – (11) yield a general model of resonant wave interactions in the presence of a strongly nonlinear response [i.e., Eq. (10)] and a generic excitation *V*(*t*) constituted by both periodic and random terms. This model can be interesting in many different contexts and in particular in nonlinear optics, where oscillators similar to (10) – (11) are at the base of the study of various types of light-matter interactions^35^,^36^. This aspect will be systematically investigated in future work. In order to highlight the simple nature of the coordinates transform (6) – (7), we cast Eqs. (1) and (9) in the form 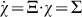, with *χ* = (*m*, *p*) and: 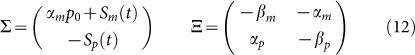
for the biological case, and *χ* = (*x*, *y*) with: 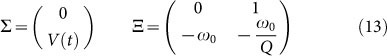
for the electric case. In absence of external sources [i.e., for *S_m_*(*t*) = 0 or *V*(*t*) = 0], Eqs. (12) – (13) possess an equilibrium point, which we indicate by (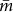, 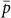) or (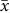, 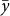). Such a fixed point is promptly calculated from 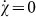 and reads 
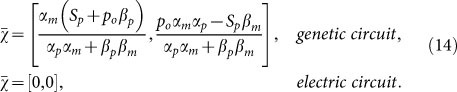
The simplicity of the coordinate transformation (6) – (7) is now clear by comparing the two expressions in Eqs. (14), which show that the RLCD system offers a simple representation where the fixed point lies in the origin of the coordinates. The scaling constant *γ*_0_ can be recast in the form 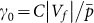, thus indicating that the divergence of *γ*_0_ for a vanishing denominator is approached when 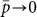 from above, i.e. when the equilibrium point is at the limit of the field of existence of *p*.

### Numerical results with sinusoidal inputs

**Figure 2:**
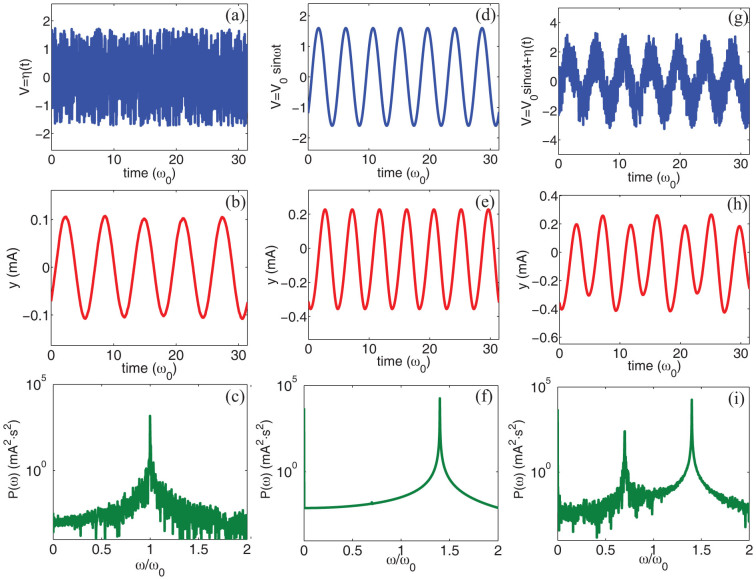
Summary of numerical results for random sinusoidal sources. (a–c) Simulations with incoherent noise *V*(*t*) = *η*(*t*), (d–f) dynamics with coherent input *V*(*t*) = *V*_0_ sin *ω_p_t* and (g–i) results from the combination of coherent and incoherent signals *V*(*t*) = *V*_0_ sin *ω_p_t* + *η*(*t*). Panels (a,d,g) show the time evolution of the input source *V*(*t*), panels (d,e,f) display the time dynamics of the current *y*(*t*) and panels (c,f,i) illustrate the current power density spectrum 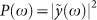. In the simulation we set *V*_0_ = 1.6 V, *T* = 10^−7^ W and *ω_p_*/*ω*_0_ = 1.4.

We studied the RLCD system via numerical integration of their respective equations of motion. Figure 2 summarizes our results in the case of a fluctuating sinusoidal periodic source *V*(*t*) = *V*_0_sin(*ω_p_t*) + *η*(*t*), being *η* a gaussian noise term with zero average 〈*η*〉 = 0 and time uncorrelated (white) variance 〈*η*(*t*)*η*(0)〉 = 2 T L *δ*(*t*). The noise variance is measured by the “temperature” *T*, which represents the total spectral power of the fluctuations. In our simulations, we investigated the effect of incoherent (Fig. 2a–c) and coherent (Fig. 2d–f) contributions separately, as well as their combination (Fig. 2g–i). In order to simulate a realistic set of electric parameters, we considered *L* = 0.01 H, *C* = 310^−13^ F, *R* = 2 Ω, *V_f_* = −0.6 V and |*V_T_*| = 600 V. This choice gives a resonant frequency *ω*_0_ = 0.58 MHz and a quality factor *Q* = 2887. In the example reported here, the source is characterized by *V*_0_ = 1.6 V, *ω_p_* = 1.4*ω*_0_, and *T* = 10^−7^ W.

**Figure 3:**
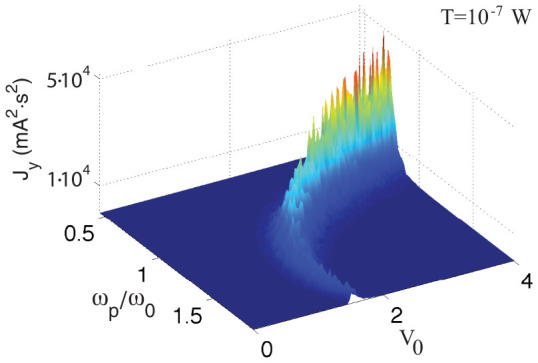
Pseudocolor plot showing the behavior of the current spectral density *J_y_* at *ω* = *ω_p_*/2 for different input frequency *ω_p_* and various voltages *V*_0_. In the simulations we set the temperature to *T* = 10^−7^ W.

Figure 2a–c displays the system dynamics when only incoherent noise *η*(*t*) is present, with temperature *T* = 10^−7^ W. At this temperature, random fluctuations have small amplitudes, and are not strong enough to generate any current in the diode. The circuit therefore behaves as a simple RLC series, with bare frequency *ω*_0_ and damping *ω*_0_/*Q*, and its main effect is to produces a “colored” noise (Fig. 2b–c). In the genetic counterpart, this implies that the system maintain a simple oscillating protein concentration *p*(*t*). When only a coherent periodic signal *V_o_* sin(*ωt*) is considered (Fig. 2d–f), with the present parameter choice the source intensity is sufficiently large to induce current in the diode. In this regime, circuits similar to that depicted in Fig. (5) have been investigated in the past, both experimentally^37^,^38^,^39^,^40^ and numerically^41^,^42^. It has been found that when the diode is characterized by a fixed junction capacity *C* and when there is no delay in the diode switching —as in the case studied here— the system displays a simple resonant behavior, without bistability, bifurcation or chaos. This behavior is confirmed in the present study: as shown in Fig. 2f, the power density spectrum 
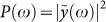
 (here 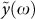 is the Fourier transform of *y*(*t*)) is particularly simple, it only shows a strong peak at the input frequency *ω* = *ω_p_* and a rectified component at *ω* = 0, which originates from the rectifying action of the diode. The very interesting dynamics is observed in Fig. 2g–i, when both coherent and incoherent sources are simultaneously present. In this case, a strong harmonic component at a new frequency, *ω* = *ω_p_*/2, appears, which represents a subharmonic of the input frequency *ω_p_*, and period doubling is observed in the dynamics (Fig. 2h–i). Quite remarkably, the strong noise resonance at *ω* = *ω*_0_ (Fig. 2c) disappeared from the dynamics. By comparing the spectra in Fig. 2i and Fig. 2f, we observe that the current power densities *P*(*ω*) at *ω* = *ω_p_* (i.e., the input pump frequency) and *ω* = 0 (i.e., the rectified component) are approximatively the same. The strong subharmonic peak at *ω_p_*/2 is therefore the result of the amplification of the noise fluctuating in the background. Stochastic contributions, in particular, are coherently amplified and constructively interact with the input signal thereby sustaining a period doubling (Fig. 2h). To further investigate this process, we calculated the intensity of the peak at *ω* = *ω_p_*/2 for different input frequencies *ω_p_* and input amplitudes *V*_0_, as reported in Fig. 3. The subharmonic peak at *ω_p_*/2 has a resonant-like intensity as a function of the pump frequency, having its maximum when *ω_p_* ≈ 3*ω*_0_/2 for low value of *V*_0_ and red-shifting at higher pump intensity. On increasing *V*_0_, the subharmonic peak maximum increases, disappearing for *V*_0_ below a *ω_p_*-dependent threshold. From Fig. 3, we observe that the efficiency of the subharmonic generation process strongly increases in the region where *ω_p_* ≈ *ω*_0_.

### Period doubling in the presence of short pulses

**Figure 4:**
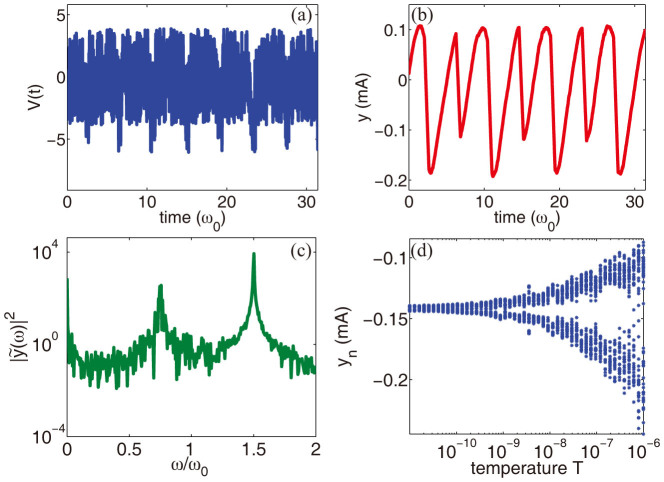
Period doubling in the presence of short pulses with time duration *δt*/*T* = 1/6. (a–c) show the case for a fixed noise *T* = 5 · 10^−7^ W, while (d) plot the behavior of the minima of the current 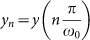 versus temperature *T*.

Periodic rhythms of biosystems are often observed as sequences of spikes, with each spike characterized by a short-living pulse. Is therefore important to investigate the occurrence of period doubling in this specific case. Figure 4a–c summarizes our results by considering square pulses of time duration *δt*/*T* = 1/6, with frequency *ω_p_* = 2*π*/*T* and amplitude *V*_0_ = 2.2 V. The noise temperature is set to *T* = 5 · 10^−7^ W. Despite the different time evolution of the source, period doubling is qualitatively identical to the one observed for sinusoidal inputs, thus witnessing the robustness and ubiquity of the phenomenon. From a quantitative perspective, the only appreciable difference is observed in the spectrum (Fig. 4c), where we report a larger bandwidth of the peak at *ω* = *ω_p_*/2 with respect to the sinusoidal case (Fig. 2i). We conclude our numerical campaign by investigating the period doubling dynamics versus the noise temperature. To this extent, we calculated the current *y* for different temperatures *T*, and we extracted the current minima *y_n_* = *y*(*nπ*/*ω_p_*), which are obtained by sampling the current at half of the input period *π*/*ω_p_*. For each temperature *T*, we averaged over 40 realization of noise and collected many sequences of *y_n_*, in order to obtain a significantly large statistic, which we reported in Fig. 4d. The figure shows that the “stochastic” period doubling observed in Fig. 2–4 does not belong to the classical bifurcation scenario of chaotic systems: no abrupt change in the period takes place, and the development of the doubling is a gentle process without any clear threshold in the control parameter (the temperature *T*). As seen from Fig. 4d, the distance between the current minima increases nonlinearly with the temperature, witnessing the strongly nonlinear nature of the process.

## Discussion

**Figure 5:**
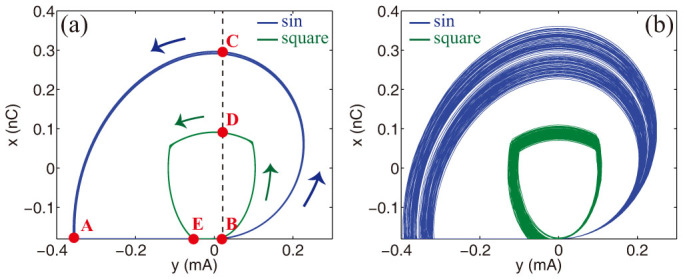
System cycle in the (*x*, *y*) plane for a sinusoidal source (blue line) and square pulses of duration *δt*/*T* = 1/6 (green line). Panel (a) shows the case without noise, panel (b) displays the dynamics and with noise at *T* = 10^−7^ W. In the simulations we set *V*_0_ = 1.6 V. The arrows in Panel (a) indicate the direction of motion along the cycles **A** – **B** – **C** – **A** and **B** – **D** – **E** – **B**.

In order to understand the role of the non linearity and, in particular, the physics of the period doubling observed in Fig. 2–4, we consider the piecewise linear model (9) and refer to Fig. 5, which displays the orbit of the system in the (*x*, *y*) plane for sinusoidal sources (blue line) and short pulses (green line) with and without noise. In absence of random fluctuations (Fig. 5a), the nonlinearity of the dynamical evolution is clearly seen as a discontinuity in the orbit tangent, taking place at points **A**, **E** (the point of the ON→OFF shift) and **B** (OFF→ON shift). When the orbit reaches points **A** or **E** the charge *x* is kept equal to *CV_f_* until the current *y* become newly positive in **B**. Following the diode in the ON state, for a sinusoidal source the orbit moves first along the semicircle (**B**-**D**), and then along the quarter of circle (**C**-**A**), which completes the cycle. In the case of square pulses, the dynamics is the same with the only difference of a smaller cycle **B**-**D**-**E**-**B**. The presence of noise does not change qualitatively the picture, but spreads out the orbits in a random fashion in both cases (Fig. 5b). Is worthwhile observing that part of the plane *x* < *CV_f_*, even for *y* > 0, is never visited (at point **A** or **E**, in fact, the charge variation 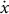 is always positive). This observation allow us to write Eqs. (9) in a more convenient form, where the switching ON-OFF conditions involve only the variable *x*: 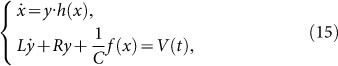
having introduced the two auxiliary functions *f*(*x*) = (*x* − *x_o_*)*θ*(*x* − *x_o_*) + *x_o_* and *h*(*x*) = 2*θ*(*x* − *x_o_*) − 1, with *x_o_* = *CV_f_* and *θ*(*x*) the Heavside step function. We can now study Eqs. (15) to investigate the origin of period doubling. To this aim, we employed a perturbation analysis from the solution of the nonlinear system in the absence of noise, i.e., for *T* = *η* = 0. Due to the smallness of the subharmonic peak with respect to the input frequency at *ω_p_* (Fig. 2i, 4c), we apply first order perturbation theory. In particular, we indicate with [*ξ*(*t*), *ζ*(*t*)] the nonlinear, noise-free solution of Eqs. (15) for *η*(*t*) = 0. Although we cannot write this solution in closed form, we know that this is strongly oscillating at *ω* = *ω_p_* in time, and lies in the curve **A-B-C-A** (or **B-D-E-B**) of Fig. 5a. In the presence of nonzero noise, we set *x*(*t*) = *ξ*(*t*) + Δ*_x_*(*t*) and *y*(*t*) = *ζ*(*t*) + Δ*_y_*(*t*). By substituting the latter expressions into Eq. (15), and retaining only the first order terms in Δ*_x_*(*t*) and Δ*_y_*(*t*), we obtain the following dynamics: 
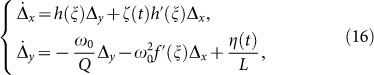
where: 
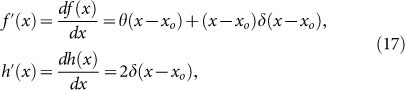
being *δ*(*x*) the Dirac-*δ* function.

**Figure 6:**
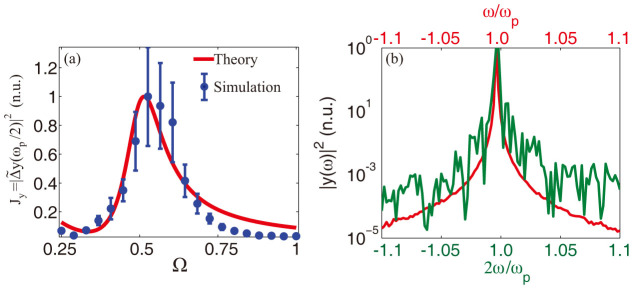
Comparison between theory and numerical simulations. Panel (a) compares the normalized subharmonic current power density 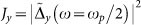 (circle markers) versus analytic theory (solid line) for different normalized frequencies Ω = *ω_p_*/2*ω*_0_. In the numerical simulations we choose *V*_0_ = 2 V, *T* = 10^−7^ W and change the input frequency in the range *ω_p_* ∈ [*ω*_0_/2, 2*ω*_0_]. For each *ω_p_* we averaged over 40 simulations to guarantee convergence of the mean value and standard deviation. Panel (b) shows the behavior of the normalized power density spectrum in the surroundings of the input frequency *ω_p_* (solid red line) and near the subharmonic peak at *ω_p_*/2 (solid green line). Spectra are calculated for a single numerical simulation with *V*_0_ = 2 V, *T* = 10^−7^ W and *ω_p_*/*ω*_0_ = 1.4.

During an orbit in the (*x*, *y*) plane, during the ON state we can distinguish between two different regions for the noise-free solution *ξ*(*t*). The first region is characterized by *ξ* + Δ*_x_* > *x_o_*, which is represented by the segment **B**-**C**-**A** (or **B**-**D**-**E**) in Fig. 5a, with points **B**, **A** (or **B**, **E**) and a small interval around them excluded. In this case *θ* = 1 and *f* = *x*, which yields *h*′ = 0 and *f*′ = 1. Equations (16) can be trivially solved in the frequency domain *ω* and the solution for the current 
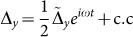
 (c.c. stands for complex conjugate) is a damped harmonic oscillator, with maximum amplitude at *ω* = *ω*_0_ and bandwidth 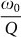: 
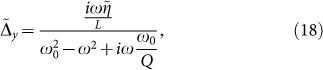
being 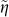 the Fourier transform of *η*(*t*). The second region of the ON state, which is the most critical, is in the vicinity of point **A** and is represented by 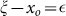, with 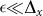. In this situation, the term Δ*_x_* leads the dynamics to continuously oscillate between the **A**-**B** (or **E**-**B**) segment (ON state) in Fig. 5a and the rest of the cycle **B**-**C**-**A** (or **B**-**D**-**E**) (OFF state). When the system switches between these two segments, the charge Δ*_x_* experiences a discontinuous dynamics originated from the term 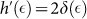, with 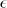 rapidly changing from zero to a small but finite value due to the noise fluctuations. The current Δ*_y_*, conversely, evolves smoothly thanks to 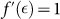. In this condition, equations of motion (16) become: 
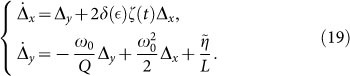
The term *ζ*(*t*) appearing in the RHS of the first equation oscillates with frequency *ω_p_*, and this imposes a resonance condition with the oscillation frequency of Δ*_x_*(*t*). In the Fourier domain, in fact, the only frequency admissible is the one where the term 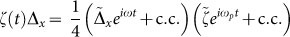 oscillates at the same frequency with Δ*_x_* and Δ*_y_*. This condition imposes phase-matching *ω_p_* ± *ω* = *ω*, which is equivalent to *ω* = *ω_p_*/2. The only oscillation that can be observed in the evolution of Δ*_x_*, Δ*_y_* is therefore at *ω* = *ω_p_*/2, cause all the frequencies that do not satisfy phase-matching are not allowed in the second region and ruled out from the dynamics. Equations (19) allow to qualitatively assess the generality of our findings with respect to the particular shape of the promoter activity function *g*(*p*). In the most general situation, the function *g*(*p*) can be expanded in Taylor series following Eq. (2). Linear terms ∝ *p*^0^ and ∝ *p* give rise, in the electric circuit representation, to linear dissipative electric components *RLC*. High order nonlinear terms ∝ *p^n^* with *n* ≥ 2, conversely, originate nonlinear dissipative terms that do not qualitatively affect the existence of the parametric resonance at *ω_p_*/2. The latter, in fact, originates from the nonlinear response of the diode that, as shown in Eqs. (6) – (7), is sustained by the mechanism of cooperative stability and the linear first order terms in the expansion of *g*(*p*). In order to solve for the current 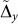, we represent *δ*(*x*) = *H* · rect(*Hx*), being rect(*x*) = *θ*(*x* + 1 − 2) − *θ*(*x* − 1/2). After taking the limit for *H* → ∞, we obtain the following solution: 
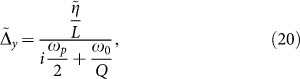
The spectral power 
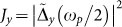
 of the current at *ω* = *ω_p_*/2 can be then obtained by combining in time the two expressions for 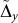 appearing in Eqs. (20) and (18) for *ω* = *ω_p_*/2 with weights 1 − *α* and *α* respectively. The latter indicates the time spent in the different region of the cycle and critically depends on the dynamics of the noise free solution (*ξ*, *ζ*). When we combine in time different spectra with different time duration, the bandwidth and the *Q*–factor of the single spectra change as well, as we are convolving the spectrum arising from an infinite signal with the Fourier transform of a box function of a finite length. We can therefore define an effective *Q*–factor 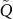, which here must be consider as a fitting parameter as it depends on the time spent by *ξ* and *η* in the different regions of the cycle. After some straightforward algebra, we obtain the following expression for the current power density: 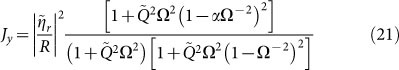
being Ω = *ω_p_*/2*ω*_0_, |*η_r_*/*R*|^2^ the current noise spectral power at the peak frequency *ω_p_*/2. Equation (21) has a nonlinear stochastic resonance at Ω ≈ 0.5, which predicts a strong noise amplification of the subharmonic *ω_p_*/2 when *ω_p_* ≈ *ω*_0_. In order to verify our theory, we calculated the behavior of *J_y_* versus Ω by a series of simulations with a sinusoidal input with a varying frequency *ω_p_*. For each *ω_p_*, we averaged over 40 realization to calculate mean value and standard deviation of *J_y_*. We then compared numerical simulations versus Eq. (21), with parameters *α* = 0.51 and 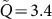 given by a nonlinear least square fit (Fig. 6a). We observed an excellent agreement between Eq. (21) and the results from numerical simulations, confirming the strongly resonant nature of the process. Equation (20) allows also to interpret the quantitative differences between the power density spectra in Fig. 2i and Fig. 4c. According to the phase matching condition imposed by Eq. (20), in fact, the bandwidth of the amplified noise is expect to match the bandwidth of coherent part the input source near *ω_p_*, where each component resonate with its subharmonics and get amplified through parametric resonance. This process is highlighted in Fig.6b, where we superimpose different part of the current power density spectrum (Fig. 6b) obtained from a numerical simulation with *V*_0_ = 1.6 V, *T* = 10^−6^ W and *ω_p_*/*ω*_0_ = 1.4. As seen in the figure, the power density of the amplified noise near *ω_p_*/2 (Fig. 6b solid green line) matches very well the spectrum of the input signal near *ω_p_* (Fig. 6b solid red line). In the case of short pulses, parametric resonance is triggered by a larger spectrum near *ω_p_*, due to the larger harmonic content of a short pulse with respect to a purely sinusoidal source, and therefore results into a larger amplified band near *ω_p_*/2.

### Beyond gene expression

The “stochastic” parametric amplification reported in this work provides a new panorama in nonlinear dynamics, whose importance goes also beyond the biological circuits introduced in this paper. A deep discussion on this subject goes clearly beyond the scope of this article, however a few interesting points can be highlighted. The nonlinear dynamics of a single non-chaotic oscillator possessing a resonant frequency at *ω*_0_, is normally observed as the generation of different higher harmonics 2*ω*_0_, 3*ω*_0_, 4*ω*_0_, …^43^. In this situation the generation of *ω*_0_/2, or equivalently period doubling, is impossible as it does not follow from any integer combination of higher harmonics. Period doubling is typically observed, in fact, in chaotic resonators and is a well-known mechanism for routing the dynamics to chaos^44^. Our works open a new interesting scenario, where period doubling can be triggered by the noise interacting with a single non-chaotic oscillator. This opens the possibility to develop applications where noise acts as an active pathway for nonlinear dynamics, enabling functionalities that would otherwise be impossible to achieve for the system. Among the possible systems that might benefit from this process, we here mention the important case provided by mid-infrared energy harvesting, where light-matter interactions are modeled by the same circuit of Fig. 1b^45^.

## Conclusions

In conclusion, we have investigated the dynamics of a elemental genetic circuit characterized by the interplay between nonlinearity and noise arising from environmental fluctuations. We showed that the circuit can be mapped into an electric analogue represented by a RLC series with a diode D. In this elementary system, nonlinearity arises naturally from the diode, which acts as a inhibitory feedback that maintains the dynamics in equilibrium. When noise and nonlinearity are considered independently in the system, we do not observe any interesting evolution, while when they exist together we observe period doubling triggered by parametric amplification of thermal noise. Parametric resonance, in particular, amplifies noise in a specific band that resonates with the coherent part of the fluctuations of the environment. This result may explain the ubiquitous period doubling phenomenon reported in different cases in biological matter, and suggests that it may be traced back to the interaction between nonlinearity and environmental fluctuations. This works also highlights the active role of noise in inhibitory processes and suggests that its modeling is key to understand the complexity of biological functions, ranging from brain activity to gene expressions.

## References

[b1] Biondi E. G. *et al.* Regulation of the bacterial cell cycle by an integrated genetic circuit. Nature 444, 899–904 (2006). 17136100 10.1038/nature05321

[b2] G. Russo M. D. B. & Sontag E. D. Global entrainment of transcriptional systems to periodic inputs. PLOS Comput. Biol. 6, 1000739 (2010). 10.1371/journal.pcbi.1000739 PMC2855316 20418962

[b3] Schmal C., Reimann P. & Staiger D. Global entrainment of transcriptional systems to periodic inputs. PLOS Comput. Biol. 9, e1002986 (2013).23555221

[b4] Garfinkel A., Spano M. L., Ditto W. L. & Weiss J. N. Controlling cardiac chaos. Science 257, 1230–1235 (1992). 1519060 10.1126/science.1519060

[b5] Etzion Y. & Grossman Y. Potassium currents modulation of calcium spike firing in dendrites of cerebellar purkinje cells. Exp. Brain Res. 122, 283–294 (1998). 9808301 10.1007/s002210050516

[b6] Mandelblat Y., Etzion Y., Grossman Y. & Golomb D. Period doubling of calcium spike firing in a model of a purkinje cell dendrite. J. Comp. Neur. 11, 43–62 (2001). 10.1023/a:1011252730249 11524577

[b7] Hellen E. H., Dana S. K., Zhurov B. & Volkov E. Electronic implementation of a repressilator with quorum sensing feedback. PLOS 8, e62997 (2013). 10.1371/journal.pone.0062997 PMC3642084 23658793

[b8] Kobayashi Y., Shibata T., Kuramoto Y. & Mikhailov A. S. Robust network clocks: Design of genetic oscillators as a complex combinatorial optimization problem. Phys. Rev. E 83, 060901–(4) (2011). 10.1103/PhysRevE.83.060901 21797294

[b9] Jia B., Gu H., Li L. & Zhao X. Dynamics of period-doubling bifurcation to chaos in the spontaneous neural firing patterns. Cogn. Neur. 6, 89–106 (2012). 10.1007/s11571-011-9184-7 PMC3253168 23372622

[b10] Rabinovich M. I., Varona P., Selverston A. I. & Abarbanel A. D. I. Dynamical principles in neuroscience. Rev. Mod. Phys. 78, 1213–1265 (2006).

[b11] Rodrigo G., Carrera j. & Jaramillo A. Computational design and evolution of the oscillatory response under light-dark cycles. Biochimie 90, 888–897 (2008). 18331846 10.1016/j.biochi.2008.02.012

[b12] Klevecz R. R. & Murray D. B. Genome wide oscillations in expression. Mol. Biol. Rep. 28, 73–82 (2001). 11931391 10.1023/a:1017909012215

[b13] Klevecz R. R., Li C. M., Marcus I. & Frenkel P. H. Collective behavior in gene regulation: the cell is an oscillator, the cell cycle a developmental process. FEBS J. 275, 2372–2384 (2008). 18410382 10.1111/j.1742-4658.2008.06399.x PMC2858570

[b14] Li C. M. & Klevecz R. R. A rapid genome-scale response of the transcriptional oscillator to perturbation reveals a period-doubling path to phenotypic change. PNAS 103, 16254–16259 (2006). 17043222 10.1073/pnas.0604860103 PMC1613231

[b15] Pfeuty B., Thommen Q. & Lefranc M. Robust entrainment of circadian oscillators requires specific phase response curves. Biophys. J. 100, 2557–2565 (2011). 21641300 10.1016/j.bpj.2011.04.043 PMC3117189

[b16] Hasegawa Y. & Arita M. Enhanced entrainability of genetic oscillators by period mismatch. J. R. Soc. Interf. 10, 20121020–(13) (2013). 10.1098/rsif.2012.1020 PMC3627117 23389900

[b17] Scott M., Hwa T. & Ingalls B. Deterministic characterization of stochastic genetic circuits. PNAS 104, 7402–7407 (2007). 17446275 10.1073/pnas.0610468104 PMC1863463

[b18] Samoilov M., Plyasunov S. & Arkin A. P. Stochastic amplification and signaling in enzymatic futile cycles through noise-induced bistability with oscillations. PNAS 102, 2310–2315 (2005). 15701703 10.1073/pnas.0406841102 PMC548975

[b19] Qian M., Shi P.-Z. & Xing J. Stochastic bifurcation, slow fluctuations, and bistability as an origin of biochemical complexity. Phys. Chem. Chem. Phys. 11, 4861–4870 (2009). 19506761 10.1039/b900335p

[b20] Shahrezaei V., Ollivier J. F. & Swain P. S. Colored extrinsic fluctuations and stochastic gene expression. Mol. Syst. Biol. 4, 196–(9) (2008). 18463620 10.1038/msb.2008.31 PMC2424296

[b21] Singh A. & Hespanha J. P. Optimal feedback strength for noise suppression in autoregulatory gene networks. Biophys J. 96, 4013–4023 (2009). 19450473 10.1016/j.bpj.2009.02.064 PMC2712194

[b22] Oyarzún D. A., Lugagne J.-B. & Stan G.-B. V. Noise propagation in synthetic gene circuits for metabolic control. ACS Synth. Biol. (2014). 10.1021/sb400126a 24735052

[b23] Levant M. & Steinberg V. Amplification of thermal noise by vesicle dynamics. Phys. Rev. Lett. 109, 268103–(5) (2012). 23368624 10.1103/PhysRevLett.109.268103

[b24] Buzsaki G. Rhythms of the Brain (Oxford University Press, New York, 2011).

[b25] Vervaeke K., Lörincz A., Nusser Z. & Silver R. A. Gap junctions compensate for sublinear dendritic integration in an inhibitory network. Science 335, 1624–1628 (2012). 22403180 10.1126/science.1215101 PMC3587282

[b26] van Vreeswijk C. & Sompolinsky H. Chaos in neuronal networks with balanced excitatory and inhibitory activity. Science 274, 1724–1726 (1996). 8939866 10.1126/science.274.5293.1724

[b27] Sompayrac L. & Maaloe O. Autorepressor model for control of dna replication. Nature 241, 133–135 (1973). 4573387 10.1038/newbio241133a0

[b28] Lenz P. & Soegaard-Andersen L. Temporal and spatial oscillations in bacteria. Nat. Microbiol. 9, 565–577 (2011). 10.1038/nrmicro2612 21760621

[b29] Chen B.-S., Hsu C.-Y. & Liu J.-J. Robust design of biological circuits: Evolutionary systems biology approach. J. Biomed. Biotech. 2011, 304236–(14) (2011). 10.1155/2011/304236 PMC3237015 22187523

[b30] Gardner T. S., Cantor C. R. & Collins J. J. Construction of a genetic toggle switch in escherichia coli. Nature 403, 339–342 (2000). 10659857 10.1038/35002131

[b31] Jenal U. & Hengge-Aronis R. Regulation by proteolysis in bacterial cells. Curr. Opin. Microbiol. 6, 163–172 (2003). 12732307 10.1016/s1369-5274(03)00029-8

[b32] Gottesman S. & Maurizi M. R. Regulation by proteolysis: energy-dependent proteases and their targets. Microbiol. Rev. 56, 592–691 (1992). 1480111 10.1128/mr.56.4.592-621.1992 PMC372890

[b33] Buchler N. E., Gerland U. & Hwa T. Nonlinear protein degradation and the function of genetic circuits. PNAS 102, 9559–9564 (2005). 15972813 10.1073/pnas.0409553102 PMC1172234

[b34] Sedra A. S. & Smith K. C. Microelectronic Circuits (Oxford, New York, 2004).

[b35] Boyd R. W. Nonlinear Optics (Academic Press, Boston, 2008).

[b36] Agrawal G. Nonlinear Fiber Optics (Academic Press, Boston, 2012).

[b37] Testa J., Perez J. & Jeffries C. Evidence for universal chaotic behavior of a driven nonlinear oscillator. Phys. Rev. Lett. 48, 714–717 (1982).

[b38] Hunt E. R. Comment on a driven nonlinear oscillator. Phys. Rev. Lett. 49, 1054–1054 (1982).

[b39] Testa J., Perez J. & Jeffries C. Testa, prez, and jeffries respond. Phys. Rev. Lett. 49, 1055–1055 (1982).

[b40] Hunt E. R. Stabilizing high-period orbits in a chaotic system: The diode resonator. Phys. Rev. Lett. 67, 1953–1955 (1991). 10044297 10.1103/PhysRevLett.67.1953

[b41] Rollins R. W. & Hunt E. R. Exactly solvable model of a physical system exhibiting universal chaotic behavior. Phys. Rev. Lett. 49, 1295–1298 (1982).

[b42] Kim C.-M., Cho C.-H. & Lee C.-S. Period-doubling bifurcation in an electronic circuit with a fast-recovery diode and square-wave input. Phys. rev. A 38, 1645–1648 (1988). 10.1103/physreva.38.1645 9900554

[b43] Nayfeh A. H. & Mook D. T. Nonlinear Oscillations (Wiley, San Francisco, 1995).

[b44] Ott E. Chaos in Dynamical Systems (Cambridge University Press, Cambridge, 1997).

[b45] Byrnes S. J., Blanchard R. & Capasso F. Harvesting renewable energy from earths mid-infrared emissions. PNAS 111, 3927–3932 (2014). 24591604 10.1073/pnas.1402036111 PMC3964088

